# Chitosan-modified ceftazidime loaded polyhydroxyalkanoates microparticles: preparation, characterization and antibacterial evaluation *in vitro*

**DOI:** 10.5599/admet.2645

**Published:** 2025-03-18

**Authors:** Anastasiya Murueva, Natalia Zhila, Alexey Dudaev, Ekaterina Shishatskaya, Tatiana Volova

**Affiliations:** 1 Institute of Biophysics SB RAS, Federal Research Center “Krasnoyarsk Science Center SB RAS”, 50/50 Akademgorodok, Krasnoyarsk 660036, Russia; 2 Siberian Federal University, 79 Svobodnyi av., Krasnoyarsk 660041, Russia

**Keywords:** Degradable polyhydroxyalkanoates, P(3HB), copolymer P(3HB-3HV-3HHх), solvent evaporation, structure and properties, antibacterial drug, release kinetics, hemolysis, keratinocyte proliferation

## Abstract

**Background and purpose:**

The use of drug delivery systems to enhance the efficacy of existing antimicrobial drugs is one of the promising approaches to combat bacterial resistance. The simultaneous presence of a polycationic biopolymer (chitosan) and an antibacterial drug (ceftazidime) in polyhydroxyalkanoates microparticles is more effective since it allows such carriers to have a more pronounced therapeutic effect. In this study, chitosan-modified ceftazidime-loaded poly(3-hydroxybutyrate-3-hydroxyvalerate-3-hydroxyhexanoate) (P(3HB-3HV-3HHх)) microparticles were prepared and investigated as a drug delivery system.

**Experimental approach:**

The obtained microparticles were characterized in terms of their particle size, polydispersity index (PDI), zeta potential, encapsulation efficiency, drug release studies *in vitro*, cytotoxicity and antibacterial properties in cell cultures.

**Key results:**

The microparticles had spherical shapes with diameters from 0.6 to 1.6 μm. The constructed chitosan-modified ceftazidime-loaded microparticles are a depot form of drug, the release of which *in vitro* is realized for a long time, without burst releases, corresponds to Korsmeyer-Peppas and Higuchi models. *In vitro* cell viability and proliferation studies on designed microparticles investigated using HaCaT (human keratinocyte skin cell lines) showed good cell proliferation. The hemolytic activity of chitosan-modified P(3HB- 3HV-3HHх) microparticles evaluated by hemolysis assay demonstrated good blood compatibility. Chitosan-modified microparticles enhanced the antibacterial activity of ceftazidime, being effective against *E. coli* and *St. aureus*.

**Conclusion:**

Thus, the obtained drug delivery systems based on PHAs and chitosan in the form of microparticles can be promising means in treating infectious skin diseases for topical use.

## Introduction

Over the past few decades, many studies have focused on developing new and effective drug delivery systems for various areas of biomedicine. Over time, this trend will increase as there is a need to reduce the cost of medical care and improve the effectiveness of existing dosage forms [1,2].

Currently, the resistance of microorganisms to antibiotics has reached alarmingly high levels worldwide. The World Health Organization (WHO) has included antimicrobial resistance in the list of 10 major global health problems [3]. Severe bacterial infections leading to sepsis are one of the most common causes of death. Therefore, more effective drugs and new approaches to antibiotic therapy are needed to prevent and combat bacterial infections [4-5]. The development of new antibiotics with broad mechanisms of action, as well as the use of delivery systems to improve the effectiveness of existing antimicrobial drugs, is a promising approach in antibacterial therapy [6,7]. Micro- and nanoparticle drug delivery systems may be one of the options for solving problems associated with the development of resistance in pathogenic microorganisms by increasing the circulation time of the antibacterial drug in the body, targeting infectious sites, reducing the toxicity of the drug, and providing protection and enhancing the antibacterial activity of existing traditional forms of antibiotics. In addition, such antibacterial drug delivery systems can improve the permeability and interaction of the drug with the bacterial membrane [2,4,8,9].

In the last decade, polyhydroxyalkanoates (PHAs) have attracted considerable interest as biomaterials for creating matrices in tissue engineering and for depositing drugs due to their biocompatibility and biodegradability, as well as the possibility of varying the physicochemical and mechanical properties of PHAs, obtaining mixtures and composites with other materials [10]. PHAs are polyesters obtained as a result of microbiological fermentation. The production of different types of PHAs can be achieved by the appropriate selection of the substrate and bacterial strain used for polymer synthesis [11]. The most common ones include the homopolymer poly-3-hydroxybutyrate (P(3HB)) and copolymers formed on its basis with different incorporation of 3-hydroxyvalerate P(3HB-3HV). Despite the excellent biocompatibility of P(3HB), high crystallinity, brittleness, and hydrophobic nature with low thermal stability limit and restrain its application in biomedicine [12]. In addition, drug delivery systems based on a single-component carrier material often result in a so-called burst release effect and are unable to sustain drug release over a long period of time. Therefore, more complex drug delivery systems consisting of two- or more-component copolymers, as well as composite carriers, are being investigated. These systems create additional barriers to drug diffusion, reducing the burst release [13].

Mixing PHAs with other natural polymers, such as polysaccharides, is a promising method for developing a new composite material with modified physicochemical properties [14,15]. One of the most common polysaccharides is chitosan, obtained by deacetylation of chitin. Chitosan is a linear polysaccharide and is of great interest in the development of drug delivery systems due to its biocompatibility, biodegradability, hydrophilicity, non-toxicity and antimicrobial activity [9,16,17]. The presence of many active groups in the polysaccharide, such as hydroxyl and amino groups, allows for interaction with P(3HB) macromolecules. The antimicrobial property of chitosan is associated with the interaction of its cationic amino groups with anions on the surface of bacterial cells, causing their death [8]. Currently, micronized forms of antibacterial drugs based on polymers of lactic acid and polycaprolactone, coated or conjugated with chitosan, are being actively developed [18-20].

Existing composite materials based on PHAs and chitosan are usually obtained in the form of films and non-woven membranes from the P(3HB) or more elastic copolymer of 3-hydroxybutyrate-4-hydroxybutyrate P(3HB-4HB) copolymer containing antibiotics, antiseptics, plant extracts, gold and silver nanoparticles, *etc*. [8,13,21]. Thus, the literature presents examples of film membranes made of P(3HB) containing vanillin and suppressing the growth of *Staphylococcus aureus*, *Shigella flexneri*, and *Salmonella typhimurium* [22]. Nanomelanin loaded into the P(3HB) membrane exerted antibacterial activity against multidrug-resistant *St. aureus* [23]. Films obtained from the copolymer of 3-hydroxyoctanoate and 3-hydroxydecanoate P(3HO-co-3HD) containing lime oil showed more pronounced antibacterial properties in the culture of gram-positive bacteria *St.aureus* compared to gram-negative *E. coli*. [24]. At the same time, there are few known examples of obtaining micro- and nanoparticles based on PHAs and chitosan-containing drugs [25-27]. The results obtained in cultures of pathogenic microorganisms demonstrated the antibacterial activity of composite microparticles made from P(3HB), P(3HB-3HV) copolymer and chitosan against *Enterococcus faecalis*, *Pseudomonas aeruginosa* [25], *Escherichia coli* and *Staphylococcus aureus* [27].

The simultaneous presence of a polycationic biopolymer (chitosan) and an antibacterial drug in PHAs microparticles is more effective since it allows such carriers to have a more pronounced therapeutic effect. However, there is a risk associated with the fact that the presence of several components in such a system, including those with antibacterial activity, can have a negative effect on blood cells and on the processes of epidermal cell proliferation at the site of the defect and inhibit tissue genesis. Therefore, when designing such microparticles intended for topical application, studies are needed to identify the effect of the polymer composition on the growth of cells involved in the regeneration of damaged tissues and the suppression of pathogens that infect wound defects.

Thus, the aim of the work was to develop a delivery system for an antibiotic (ceftazidime) in the form of chitosan-modified PHAs-based microparticles and to study their characteristics, drug release kinetics and their biological activity with respect to blood cells, epidermal cells and pathogenic microorganisms *in vitro*.

## Experimental

### Materials

The PHA samples were synthesized by the wild-type strain *Cupriavidus necator* B-10646 from the collection of the Institute of Biophysics SB RAS. The method of polymer synthesis, composition and properties, and research methods were described in detail earlier [28,29]. The substrates for the synthesis of PHA samples were fructose (Sigma-Aldrich, USA, purity 99 %), myristic acid (purity 97 %, Acros Organics, Brussels, Belgium), oleic acid (purity 98 %, EKOS-1, Staraya Kupavna, Russia), palm oil (Malaysia, purchased from a local supermarket). Fructose was added into the medium at a concentration of 10-15 g/L, the other substrates were added into the medium at a concentration of 15 g/L. The characteristics of the PHA samples synthesized on various substrates are presented in Table 1.

**Table 1.** Physicochemical properties of PHAs synthesized by the wild-type strain *Cupriavidus necator* B-10646 on various substrates

Substrates	PHAs monomer content, mol.%			*M*_w_ / kDa	*M*_n_ / kDa	Đ	*C*_x_ / %	*T*_melt_ / °C	*T*_degr_ / °C
	3HB	3HV	3HHх						
Fructose	100	0	0	450	160	2.8	73	175	290
Myristic acid	100	0	0	365	99	3.7	69	172	274
Palm oil	97.2	1.9	0.9	670	86	3.6	67	172	278
Oleic acid	97.96	2.02	0.02	485	59	4.2	68	174	280

*M*_w_ - average molecular weight; *M*_n_ - number average molecular weight; Ð – polydispersity (Ð = *M*_w_/*M*_n_); *C*_x_ - degree of crystallinity; *T*_melt_ - melting point; *T*_degr_ - degradation temperature

Chitosan (*M*_w_ 160 KDa with a deacetylation degree > 80 %) was obtained from Xi'an Ltd., China. The drug used was the third-generation broad-spectrum cephalosporin antibiotic Protozidime^®^ (ceftazidime (CEF)) (Pharmsintez JSC, Russia).

### Preparation of polyhydroxyalkanoates microparticles

Polymeric microparticles were prepared using a solvent evaporation method. Briefly, a weighed amount of PHAs (200 mg) was dissolved in 10 mL of dichloromethane. Farther, this solution was added dropwise into 100 mL of an aqueous solution 0.5 % PVA under stirring. The obtained emulsions were mechanically stirred at 24000 rpm for 5 min (IKA Ultra-Turrax T25 digital high-performance homogenizer, Germany). All w/o emulsions were mixed mechanically for 24 h until the solvent completely evaporated. The resulting aqueous dispersions were centrifuged to collect the microparticles (10, 000 rpm, 20 min). After this, the microparticles were washed four times in distilled water and freeze-dried in Alpha 1–2 LD plus (Christ, Germany).

Ceftazidime-loaded PHA microparticles were prepared using the oil-in-water solvent emulsion evaporation method with minor modifications. The ceftazidime (20 mg) was dissolved in 1 ml of DMSO and added to the 0.2 g P(3HB-3HV-3HHх) solution in dichloromethane. Then, the obtained solution was added to 0.5 % (w/v) PVA solution at stirring at 24,000 rpm (IKA Ultra-Turrax T25 digital high-performance homogenizer, Germany). The emulsion was stirred at 700 rpm, at room temperature, until the organic solvent was completely evaporated. Centrifugation and washing conditions were similar to those described earlier.

### Preparation of chitosan-modified polyhydroxyalkanoates microparticles

Modifications to the solvent evaporation method were used to prepare chitosan-modified microparticles. The emulsion contained 0.2 g P(3HB-3HV-3HHх) in 10 mL. The aqueous phase consisted of chitosan and PVA solution, both prepared using 1 vol.% acetic acid. The amount of chitosan varied from 50 to 200 mg. All remaining procedures are the same as above. The method described above was also used to load ceftazidime into chitosan-modified microparticles. Depending on the composition of drug-loaded formulations, the resulting microparticles were denoted as P(3HB-3HV-3HHх)-CEF and chitosan-modified microparticles as P(3HB-3HV-3HHх)-CS-CEF (Table 2).

**Table 2.** Summary of chitosan-modified microparticles used in this work

Abbreviation	Content, mg		
	P(3HB-3HV-3HHх)	Chitosan	Ceftazidime
P(3HB-3HV-3HHх)-CS_1_	200	50	-
P(3HB-3HV-3HHх)-CS_2_	200	100	-
P(3HB-3HV-3HHх)-CS_3_	200	200	-
P(3HB-3HV-3HHх)-CEF	200	-	20
P(3HB-3HV-3HHх)-CS_1_-CEF	200	50	20
P(3HB-3HV-3HHх)-CS_2_-CEF	200	100	20
P(3HB-3HV-3HHх)-CS_3_-CEF	200	200	20

### Characterization of the microparticles

Scanning electron microscopy (SEM, S-5500, Hitachi, Tokyo, Japan) was used to observe the surface morphology of microparticles. The microparticles were sputter-coated with platinum using an electrical potential of 2.0 kV at 25 mA for 6 min with a sputter coater K550X (Emitech, Quorum Technologies Ltd., UK).

The particle size and polydispersity index (PDI) of microparticles were measured using the dynamic light scattering technique on the Zetasizer Nano (Zetasizer Nano ZS, Malvern Instruments, Malvern, U.K.). Freshly prepared suspension of samples was measured after dilution with bidistilled water. Zeta potential (ζ potential) was determined using the same instrument in a disposable folded capillary zeta cell at 25 °C. For statistical analysis, all samples were measured in triplicate, and the average values and standard deviation of the measurements were calculated.

Polymer content in prepared microparticles was analyzed with a GC-MS (7890/5975C, Agilent Technologies). Microparticles were subjected to methanolysis in the presence of sulfuric acid, methanol and chloroform at 80 °C for 2 h and 40 min. Benzoic acid was used as an internal standard to determine the polymer content in microparticles. Monomer units were identified based on their retention times and mass spectra.

IR spectroscopy with NICOLET 6700 FT-IR spectrometer (Thermo Scientifc, U.S.) was used to study the nature of the interaction between the copolymer P(3HB-3HV-3HHх), chitosan and drug. IR spectra were taken in the 400 to 4000 cm^-1^ range with the Smart Orbit accessory by the attenuated total reflection (ATR) technique. The absolute value of the maximum permissible error is ±0.01 cm^-1^.

### Determination of the drug content and encapsulation efficiency

The encapsulation efficiency of ceftazidime loaded into P(3HB-3HV-3HHx) microparticles and chitosan-modified P(3HB-3HV-3HHx) microparticles was determined using the method described previously [30]. The ceftazidime amount was estimated in supernatants after collecting microparticles by centrifugation. The amount of ceftazidime loaded into the polymer microparticles was determined spectrophotometrically (Uvicon 943, Italy) by measuring the UV-Vis absorbance at 256 nm using pre-built calibration graphs. The experiment was carried out in triplicates. The ceftazidime content was calculated from the calibration curves and expressed as encapsulation efficiency (EE). EE was defined as the percentage of the mass of the drug in microparticles (*M*_m_) compared to the initial mass of the drug (*M*_i_), as shown in Equation (1).



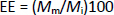

(1)


### In vitro drug release

The *in vitro* drug release was carried out in phosphate buffer solution pH 7.4 at 37 °C. The microparticles were initially sterilized by UV radiation for 40 min and placed in a sterile centrifuge tube containing 5 ml of phosphate-buffered saline (PBS, pH 7.4). P(3HB-3HV-3HHх) microparticles and chitosan-modified microparticles P(3HB-3HV-3HHх) were precipitated by centrifugation (10,000 rpm, 10 min). Two milliliters of the release medium were taken out at the predetermined time, and fixed volume was maintained by adding 2 mL of fresh PBS buffer. This experiment was performed in triplicate, and the average value with standard deviation was plotted at different time points. The results were then plotted as the cumulative release. The data obtained was then fitted into different release kinetics models: zero order, first order, Hixson-Crowell release model, Higuchi release model and Korsmeyer-Peppas release model [31].

### Hemolytic activity studies

Hemocompatibility of the microparticles was examined by conducting a hemolysis assay and blood coagulation tests [32]. The study protocol was reviewed and approved by the Ethics Committee of the Siberian Federal University. The study was designed and conducted in accordance with ISO 10993-4 [33]. Blood was collected from healthy donors from the cubital vein using an atraumatic protocol. Blood samples were collected in a BD Vacutainer pre-filled with EDTA (final concentration 1.6 mg/mL, BD, USA) for blood preanalysis and immediately gently shaken to mix the anticoagulant with the blood.

Hemolytic activity was assayed using a suspension of erythrocytes from anticoagulated blood separated from plasma by centrifugation (10 min at 3000 rpm) and subsequently washed with 0.9 % NaCl saline. 2 vol.% erythrocyte suspension was incubated with the microparticles at final concentrations of 0.05, 0.1, 0.25 and 0.5 mg/mL in PBS with pH 7.4, as well as in ultrapure water and saline as positive and negative controls, respectively. All samples were incubated at 37 °C for 2 and 24 h to study the effects of short-term and long-term exposure. In each period, the samples were centrifuged for 10 min at 3000 rpm to separate the cell mass and the test particles; the supernatant was used to measure the concentration of free hemoglobin by spectrophotometry at a wavelength of 550 nm using a Bio-Rad 680 microplate reader (Bio-Rad LABORATORIES Inc., USA). The hemolysis coefficient was calculated using the following equation 2:



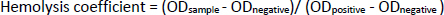

(2)


where OD_sample_ is the optical density of the supernatant in the experimental samples; OD_negative_ is the optical density of the supernatant in the negative control; OD_positive_ is the optical density of the supernatant in the positive control.

The obtained anticoagulated blood with EDTA was added to the particles at concentrations of 1, 2.5, 5, 10 and 20 mg/mL, incubated on a thermoshaker-incubator for plates and recalcified with CaCl_2_ (Sigma-Aldrich) at a concentration of 25 mM at 100 rpm to form a thrombus. The test was carried out at a temperature of 37 °C for 2 hours. The resulting thrombi were washed with 0.9 % saline solution of NaCl to remove weakly attached cells. The thrombi were fixed with glutaraldehyde at room temperature overnight, then dehydrated and dried to the critical point and weighed.

### Biocompatibility of microparticles in epithelial cell culture

The cellular biocompatibility test was performed in the human keratinocyte culture HaCaT (CLS Cell Lines Service GmbH, Germany). The wells of 96-well culture plates (Corning Costar, USA) were pre-seeded with keratinocytes at a density of 1.5×10^4^ cells/cm^2^. Cultivation was carried out according to the standard technique in DMEM medium: 4.5 g/L glucose, 4 mM L-glutamine, 1.5 g/L NaHCO_3_, 1.0 mM sodium pyruvate with the addition of 10 % fetal calf serum and 1 % antibiotic-antimycotic solution (Gibco, Invitrogen) in a humid atmosphere (5 % CO_2_) at a temperature of 37 °C in a CO_2_ incubator (New Brunswick Scientific, USA) for 24 h until 80 % confluence was achieved. In the wells with cells, the nutrient medium was replaced with a nutrient medium with the studied microparticles at a maximum concentration of 20 mg/mL for complete coverage of the well area and maximum contact. After a further 24 h, live and dead cells were analyzed using the ReadyProbes™ Cell Viability Imaging Kit, Blue/Green (Thermo Fisher Scientific) consisting of NucBlue^®^ Live (Hoechst 33342), which stains the nuclei of all cells, and NucGreen^®^ Dead, which stains only the nuclei of dead cells with compromised plasma membrane integrity. Images were acquired immediately using a Leica DMI8 inverted microscope with the corresponding LAS X software version 3.7.4.23463 with the appropriate filters. Five images were acquired per sample. Results are expressed as the mean percentage of viable cells: number of blue cells/(number of blue cells + number of green cells)×100. The metabolic activity of cells was assessed in the MTT assay based on the reduction of 3-(4,5-dimethylthiazol-2-yl)-2,5-diphenyltetrazolium bromide (Sigma) by cellular dehydrogenases to violet crystals of MTT formazan. The medium in the wells with cultured cells was replaced with a freshly prepared medium containing 5% MTT solution. After 4 h of incubation, the plates were unscrewed; the culture medium was replaced with DMSO to dissolve the formed formazan crystals. After 30 min, the colored solutions were transferred to a 96-well plate and the optical density was measured at a wavelength of 550 nm on a Bio-Rad 680 microplate reader (Bio-Rad LABORATORIES Inc., USA).

### Microbiological evaluation

The antibacterial activity of P(3HB-3HV-3HHx) and chitosan-modified P(3HB-3HV-3HHx) microparticles containing ceftazidime against model gram-positive bacteria *Staphylococcus aureus* and gram-negative bacteria *Escherichia coli* was assessed using the disk diffusion method [34]. A sterile cotton swab soaked into *St. aureus* or *E. coli* strains corresponding to a 0.5 McFarland standard solution was used to inoculate the surface of the agar plate. The concentration of encapsulated ceftazidime when microparticles were introduced into the bacterial culture as a suspension was 0.8 mg. Plates were incubated at 37 °C for 24 h and then the inhibition zones were measured, experiments were performed in triplicates.

### Statistics

Statistical analysis was carried out using Microsoft Excel and showed the mean standard deviation using the student’s *t*-test. The difference with *P* values < 0.05 was considered statistically significant.

## Results and discussion

### Characteristics of microparticles

In order to select samples of PHAs from which it is possible to obtain small-sized particles, comparative studies of the structure and size characteristics of microparticles obtained by the emulsion method were carried out for subsequent modification of the surface of the microparticles with chitosan. It was previously shown that the method of obtaining microparticles, the emulsion mixing rate and the chemical composition of the polymer have a significant effect on the size characteristics of the particles [35].

In this work, PHAs microparticles synthesized on different types of substrates were obtained. As shown earlier, the type of substrate used affected the physicochemical properties of the polymers and their monomer composition (Table 1). The use of fructose and myristic acid as a carbon source ensured the synthesis of a homopolymer, P(3HB), while palm oil and oleic acid allowed us to obtain a three-component copolymer P(3HB-3HV-3HHx) with a comparable content of 3HV and 3HHx monomers (Table 1). A detailed description of the synthesis conditions and the physicochemical characteristics of the polymers are described in detail earlier [28,29]. PHA microparticles of various chemical compositions were obtained using the one-step emulsification method. Thus, the yield of finished PHA microparticles was about 80 % (Table 3).

**Table 3.** Characteristics of PHAs microparticles obtained on different C - substrates

Substrates	PHAs monomers content, mol. %			Yield, %	Particle size, μm	PDI	Zeta potential, mV
	3HB	3HV	3HHх				
Fructose	100	0	0	82.3	1.57±0.02	0.26±0.03	-15.3±1.3
Myristic acid	100	0	0	83.5	1.37±0.06	0.27±0.02	-21.0±1.0
Palm oil	97.2	1.9	0.9	74.5	0.96±0.01	0.10±0.06	-18.9±0.6
Oleic acid	97.96	2.02	0.02	80.5	0.62±0.03	0.08±0.02	-16.6±1.4

**Figure 1.**
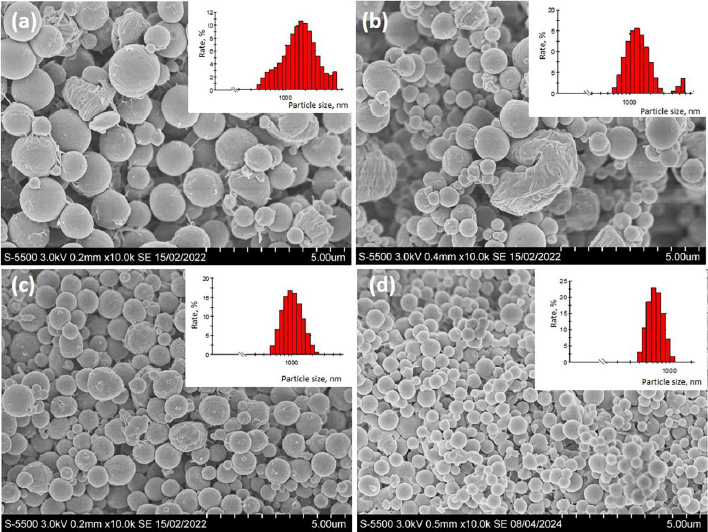
SEM images and size distribution of microparticles obtained by the emulsion method from polymer synthesized on: (a) fructose, P(3HB); (b) myristic acid; (c) palm oil P(3HB-3HV-3HHx); (d) oleic acid P(3HB-3HV-3HHx). The bar is 5 μm

It was shown that the chemical composition of the polymer affected the morphology and size of the microparticles. Analyzing the SEM images of the microparticles shown in Figure 1, one can note the presence of deformed particles obtained from P(3HB) was noted.

Microparticles obtained from P(3HB- -3HV-3HHx) copolymers had a regular spherical shape and were less heterogeneous in size – the polydispersity index varied from 0.08 to 0.27 (Table 3).

The average diameter of microparticles obtained from P(3HB-3HV-3HHx) copolymers was 1.5-2 times smaller than those obtained from P(3HB). The zeta potential of the obtained PHAs microparticles did not differ significantly and varied from -15.3 to -21 mV.

According to the literature, particles with a zeta potential of -10 to +10 mV are considered neutral, while particles with a zeta potential greater than +30 mV or less than -30 mV are strongly cationic and strongly anionic, respectively. In this regard, particles with high absolute zeta potential values are considered more stable due to the repulsion between them [36]. Values of zeta potential between -5.0 and -15.0 mV are in the flocculation boundary region, while values between -5.0 and -3.0 mV are the maximum flocculation region of the system. When studying the results obtained for PHAs microparticles synthesized on various substrates, it is evident that the zeta potential values are above the flocculation limit. This demonstrates the stability of P(3HB) and P(3HB-3HV-3HHх) microparticles. The use of the P(3HB-3HV-3HHх) copolymer obtained using oleic acid as a substrate made it possible to obtain microparticles of more uniform size with a minimum average diameter of 0.62±0.03 μm and a zeta potential value of -16.6±1.4 mV, indicating the stability of the microparticles obtained. Therefore, this copolymer was chosen for further work to obtain chitosan-modified microparticles.

**Figure 2.**
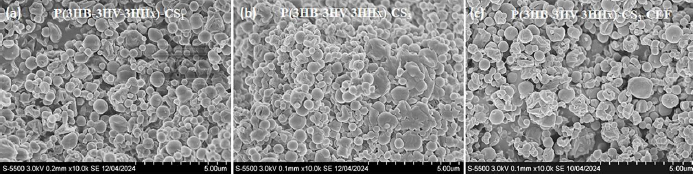
SEM images of chitosan-modified P(3HB-3HV-3HHx) microparticles: (a) P(3HB-3HV-3HHx)-CS_1_; (b) P(3HB-3HV-3HHx)-CS_3_; (c) P(3HB-3HV-3HHх)-CS_3_-CEF. The decoding of analyzed microparticles is presented in Table 2. The bar is 5 μm.

Chitosan-modified P(3HB-3HV-3HHх) microparticles were obtained with a modified one-step emulsification method. The SEM micrographs show that the chitosan-modified P(3HB-3HV-3HHх) microparticles had a heterogeneous structure with agglomerates and deformed particles (Figure 2).

A similar morphology of composite particles from P(3HB-3HV) with chitosan was observed earlier in [25]. The formation of microaggregates is shown for micro- and nanoparticles of chitosan [37,38], as well as for composite particles of polylactide with chitosan [18,39], since an increase in the concentration of chitosan in composites leads to an increase in the number of hydrogen bonds between chitosan molecules and electrostatic interactions with organic salts.

It was shown that with increasing chitosan content, the sizes of microparticles increased from 0.72±0.01 to 0.88±0.03 μm. At the same time, loading the microparticles with ceftazidime did not significantly affect their sizes. The polydispersity index (PDI), which provides information on the homogeneity of the particle size distribution in a specific system, reflects the quality of particle dispersion. According to the literature, most studies indicate that PDI values less than 0.3 in micro- and nanoparticle systems are considered as optimal [40]. The PDI value from 0.20 to 0.36, characteristic of chitosan-modified microparticles, indicates that the addition of chitosan to the P(3HB-3HV-3HHx) copolymer significantly increased the heterogeneity of the microparticles compared to P(3HB-3HV-3HHx) microparticles (Tables 3 and 4).

**Table 4.** Characteristics of chitosan-modified P(3HB-3HV-3HHx) microparticles

Composition of microparticles	Yield, %	Chitosan content in mps*,%	Particle size, μm	PDI	Zeta potential, mV	EE, %
P(3HB-3HV-3HHх)-CS_1_	81.3	4.7	0.72±0.01	0.21±0.03	34.0±1.57	-
P(3HB-3HV-3HHх)-CS_2_	80.0	8.8	0.78±0.06	0.31±0.06	51.9±1.80	-
P(3HB-3HV-3HHх)-CS_3_	84.6	18.4	0.88±0.03	0.36±0.03	61.5±2.68	-
*Ceftazidime loaded microparticles*						
P(3HB-3HV-3HHх)-CEF	82.0	-	0.67±0.05	0.20±0.02	-14.7±0.36	18.0
P(3HB-3HV-3HHх)-CS_1_-CEF	82.5	-	0.70±0.02	0.30±0.04	23.7±2.66	15.5
P(3HB-3HV-3HHх)-CS_2_-CEF	85.6	-	0.74±0.01	0.28±0.02	58.6±0.27	23.0
P(3HB-3HV-3HHх)-CS_3_-CEF	83.0	-	0.84±0.02	0.34±0.02	58.2±0.23	36.0

*mps - microparticles. The decoding of analyzed microparticles is presented in Table 2.

The zeta potential values of P(3HB-3HV-3HHx)-CS microparticles and P(3HB-3HV-3HHx)-CS-CEF reached positive values, indicating that some of the amino groups of the chitosan molecules were located on the surface of the particles. With the increase in the chitosan content, the absolute values of the zeta potential increased (Table 4), implying an increase in the system's stability.

The obtained results indicate that chitosan was successfully coated on the surface of P(3HB-3HV-3HHx) microparticles. An additional evaluation criterion indicating the successful coating of the microparticles with chitosan is the data obtained by gas chromatography. Thus, depending on the initial concentration of chitosan, its actual content in the obtained microparticles varied from 4.7 to 18.4 % (Table 4).

The encapsulation efficiency of ceftazidame in P(3HB-3HV-3HHх)-CEF and P(3HB-3HV-3HHх)-CS-CEF microparticles was comparable – 15 to 18 %. Increasing the chitosan content in the microparticles positively affected EE and reached 36 % for P(3HB-3HV-3HHх)-CS_3_-CEF microparticles (Table 4). It should be added that the content of active ingredients loaded into the particles is an important factor since a higher loading allows for the use of a smaller amount of microparticles for a given dose.

**Figure 3.**
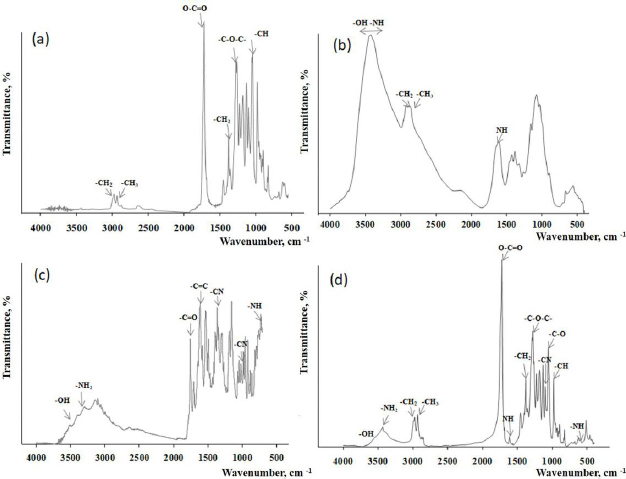
IR spectra: (a) P(3HB-3HV-3HHx), (b) chitosan, (c) ceftazidime, (d) P(3HB-3HV-3HHх)-CS-CEF microparticles

To analyze the interaction between the polymer components, IR spectra of chitosan, P(3HB-3HV-3HHх) copolymer, ceftazidime antibiotic and P(3HB-3HV-3HHх)-CS-CEF microparticles were recorded (Figure 3).

The main bands characterizing P(3HB-3HV-3HHх) correspond to the stretching vibrations of the carbonyl group of the ester (O–C=O) at a frequency of ~1720 cm^–1^. The stretching vibrations of the C–O–C group correspond to the band at ~1278 cm^−1^ in the IR spectrum. There are absorption bands of CH_3_ and CH_2_ groups (~2974, ~22931 cm^-1^); the absorption band at ~1378 cm^-1^ is related to the deformation vibrations of the CH_2_ groups; the medium-intensity band at ~1059 cm^-1^ is related to the stretching vibrations of the –C–O– bond. The band at ~978 cm^-1^ is related to the deformation vibrations of the –CH groups.

The spectrum of chitosan contains bands due to stretching vibrations of OH groups in the range from ~3750 to ~3000 cm^–1^, which overlap with the stretching vibrations of the N–H bond, and bands corresponding to stretching vibrations of the bond in the –CH_2_ (~2920 cm^–1^) and –CH_3_ (~2875 cm^–1^) groups. The band at ~1600 cm^−1^ represents the N-H deformation peak from the glucosamine unit, and the band at ~1100 cm^−1^ represents the–C–O–C– stretching [41]. The IR spectrum of ceftazidime has characteristic absorption bands in the region of 3660-3250 cm^-1^ (deformation vibrations of the bound N-H groups), which overlap with the stretching vibrations of the NH_2_ group (3250 cm^-1^); 1750 cm^-1^ (carbonyl group C=O), 1529 to 1600 cm^-1^ (deformation vibrations in the aromatic ring C=C); 1350 to 1300 cm^-1^ (axial vibrations of the C-N bond) and 1680 to 1630 cm^-1^ (stretching vibrations of C=O). The analysis of the obtained spectrum of ceftazidime is consistent with that presented earlier in other work [42].

In the spectra of P(3HB-3HV-3HHх)-CS-CEF microparticles, all the absorption bands characteristic of these substances are present without noticeable shift. The characteristic peaks of chitosan and ceftazidime were observed in the FT-IR spectrum of CS-modified P(3HB-3HV-3HHх)- microparticles, which indicated that chitosan successfully modified P(3HB-3HV-3HHх) microparticles. It should be noted that in the spectra of P(3HB-3HV-3HHх)-CS-CEF microparticles, there are no new peaks or disappearance of the original peaks, which indicates that the surface modification and loading with the antibiotic is by physical adsorption. A similar interaction of the components included in the micro-nanoparticles was noted in the work of Gaur [41]. The absence of chemical interaction between the polymer and drugs was also shown in the example of non-woven membranes and films with antibacterial drugs [43].

### Dynamics of drug release from microparticles

The drug release from polymer microparticles depends on the physicochemical properties of the material, as well as on the chemical structure of the drugs [44]. It is known that PHAs are practically not hydrolyzed in physiological buffer solutions in the absence of enzymes and cells. Therefore, the drug release rate in physiological buffer solutions that do not contain enzymes or cells will depend on the diffusion of the drug and not on the degradation of the polymer base [45].

**Figure 4.**
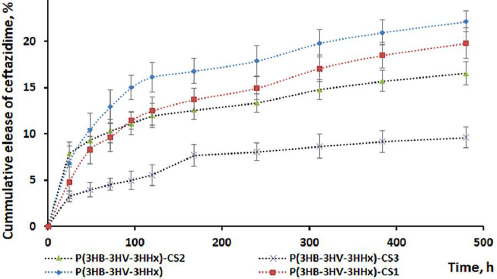
*In vitro* ceftazidime release from P(3HB-3HV-3HHх)-CEF and P(3HB-3HV-3HHх)-CS-CEF microparticles in PBS pH 7.4. Data are presented as mean value ± standard deviation, *n* = 3.

One of the objectives of this study was to investigate the effect of chitosan on the dynamics of the antibacterial drug release. For this purpose, a model medium simulates the pH of normal human blood (phosphate buffered saline pH 7.4) (Figure 4).

The release profiles of ceftazidime from P(3HB-3HV-3HHх) and chitosan-modified P(3HB-3HV-3HHх) microparticles demonstrate a 2-phase character. In the first stage, the concentration of ceftazidime in the buffer solution increased, and starting from 96 to 120 hours, the curves reached a plateau and the release rate decreased. Although the release profiles of the antibiotic from all studied microparticle samples were similar, the highest yield of ceftazidime is characteristic of unmodified P(3HB-3HV-3HHх) microparticles. Thus, the cumulative release of ceftazidime from unmodified microparticles reached 10.5 % after 48 h due to the so-called burst release effect. Such an effect is undesirable since it can decrease the efficiency of the developed system and cause side effects in the human body.

Starting from the end of the 5th day (120 h) and until the end of the observation, the intensity of ceftazidime release from P(3HB-3HV-3HHх) microparticles decreased, while the total yield of the antibiotic was 22±1.12 %. The modification of the microparticle surface with chitosan led to a decrease in the intensity of the antibiotic release. It was noted that the rate of ceftazidime release decreased with an increase in the chitosan content on the microparticle surface. Thus, the total release of ceftazidime by the end of the experiment from chitosan-modified microparticles was 20±1.67 and 16.5±1.22 % from P(3HB-3HV-3HHх)-CS_1_-CEF and P(3HB-3HV-3HHх)-CS_2_-CEF, respectively. At the highest chitosan content on the surface of microparticles (P(3HB-3HV-3HHх)-CS_3_-CEF), a pronounced decrease in the intensity of the antibiotic release was observed. Thus, after 48 hours, the antibiotic release did not exceed 4 %, and by the end of the experiment, it was 9.6±1.13 %. A similar tendency to decrease the drug release rate when the surface of PLGA microparticles was coated with chitosan was observed in a number of studies [18,20,41]. Using the example of PHAs microparticles with the addition of chitosan cross-linked with glutaraldehyde in different concentrations, the possibility of varying the release rate of piroxicam and ketoprofen was demonstrated [26].

To describe the mechanism of ceftazidime release from P(3HB-3HV-3HHх) and chitosan-modified P(3HB-3HV-3HHх) microparticles, five mathematical models were used: the zero-order model, the first-order model, the Higuchi model, the Hickson-Crowell model, and the Korsmeyer-Peppas model. Based on these mathematical models, the values of the approximation reliability coefficients (*R*^2^) were calculated (Table 5).

**Table 5.** The values of *R*^2^ from release kinetic models fitting on the in vitro drug release data of ceftazidime microparticles in phosphate buffer pH 7.4.

Composition of microparticles	*R* ^2^				
	Zero order	First order	Higuchi	Hickson-Crowell	Korsmeyer-Peppas*
P(3HB-3HV-3HHх)-CEF	0.82	0.89	**0.96**	0.94	**0.98** (*n*=0.08)
P(3HB-3HV-3HHх)-CS_1_-CEF	0.72	0.93	**0.98**	0.94	**0.99** (*n*=0.10)
P(3HB-3HV-3HHх)-CS_2_-CEF	0.67	0.91	**0.98**	0.90	**0.98** (*n*=0.02)
P(3HB-3HV-3HHх)-CS_3_-CEF	0.81	0.84	**0.99**	0.89	**0.95** (*n*=0.05)

The highest values of the approximation reliability coefficient (*R*^2^) for each of the studied samples are highlighted in bold.

According to the data in Table 5, the kinetics of ceftazidime efflux from P(3HB-3HV-3HHх) and chitosan-modified P(3HB-3HV-3HHх) microparticles can be described using two models (Higuchi and Korsmeyer-Peppas). This is confirmed by high *R*^2^ coefficients exceeding 0.95. According to the Korsmeyer-Peppas model, values of n of 0.5 or less correspond to the diffusion nature of the release of drugs and obey Fick's laws [46].

In general, it was shown that the release of ceftazidime from chitosan-modified P(3HB-3HV-3HHx) microparticles proceeds smoothly and depends on the chitosan content on the microparticle surface. At the same time, a decrease in the so-called "burst release effect" was observed with an increase in the chitosan content on the microparticle surface. The results indicated that modification of the surface of PHA microparticles with chitosan allows for the variation of the drug release rate.

### Hemolytic activity of microparticles

Currently, the literature contains conflicting data regarding the hemocompatibility of chitosan, which depends on a number of factors, such as the source of isolation and the method of obtaining the polysaccharide, molecular weight, and the degree of deacetylation [47,48]. In turn, a series of studies have confirmed the bio- and hemocompatibility of materials based on PHA [49,50].

In this work, a primary assessment of the hemolytic activity of P(3HB-3HV-3HHx) and P(3HB-3HV-3HHx)-CS microparticles was carried out depending on the concentration of microparticles and the content of chitosan on the surface of microparticles during interaction with human blood cells *in vitro*.

**Figure 5.**
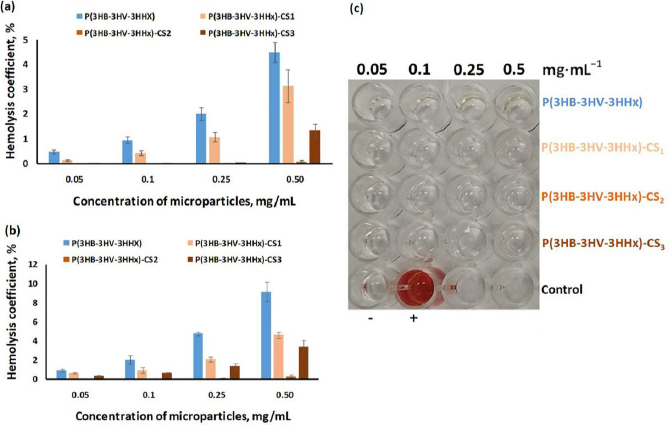
Hemolytic activities (percentage of hemolysis) of P(3HB-3HV-3HHх) and chitosan-modified P(3HB-3HV-3HHх) microparticles for 2 (a) and 24 h (b) as a function of microparticles concentrations; Photograph of the supernatant for measuring optical density after contact with the microparticles for 24 h (с). The decoding of analyzed microparticles is presented in Table 2. Data are presented as mean value ± standard deviation, *n* = 6.

In this work, the primary assessment of the hemolytic activity of P(3HB-3HV-3HHх) and P(3HB-3HV-3HHх)-CS microparticles was performed depending on microparticles concentration and the chitosan content during interaction with human blood cells *in vitro*. After 2 h, the hemolysis coefficients (Figure 5 a,b) of all samples were significantly below 5 % (risk coefficient described in the ISO TR 7405 standard [51]. After 24 hours, the hemolytic activity of less than 5 % remained for chitosan-modified P(3HB-3HV-3HHх) microparticles at concentrations of 0.05-0.25 mg/mL, and was maintained at this level with an increase in concentration to 0.50 mg/mL. The lowest value of the hemolysis coefficient was demonstrated by P(3HB-3HV-3HHх)-CS_2_ microparticles.

The hemolytic activity of P(3HB-3HV-3HHх) microparticles in concentrations from 0.05 to 0.25 mg/mL was comparable to chitosan-modified P(3HB-3HV-3HHх) microparticles (the hemolysis coefficient did not exceed 5%). With an increase in the concentration of P(3HB-3HV-3HHx) microparticles to 0.50 mg/mL, the hemolysis coefficient slightly exceeded the risk coefficient and was 9 %.

Figure 5 b shows a photograph of the supernatant of blood cells 24 h after incubation with P(3HB-3HV-3HHx) and chitosan-modified P(3HB-3HV-3HHx) microparticles. All studied samples of microparticles in concentrations from 0.05 to 0.5 mg/mL are characterized by the absence of obvious release of hemoglobin from erythrocytes, one of the indicators of high biocompatibility of microparticles in blood.

**Figure 6.**
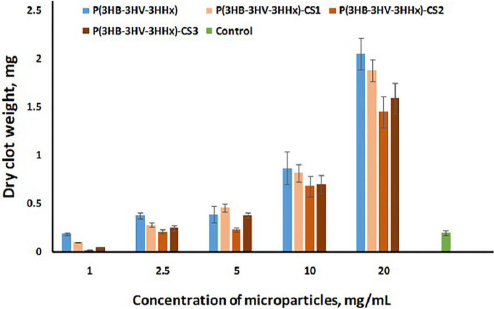
Mass of dried thrombi after decalcification of anticoagulated blood upon interaction with different concentrations of microparticles. The decoding of analyzed microparticles is presented in Table 2. Data are presented as mean value ± standard deviation, *n* = 6.

There is data in the literature that the interaction of free amino groups of chitosan with blood plasma proteins and/or blood cells can cause a thrombogenic and/or hemolytic reaction since this polysaccharide can activate both complements and blood coagulation systems [48]. The mechanism of interaction between blood and chitosan is associated with the adsorption of plasma proteins with subsequent adhesion and activation of platelets, which leads to thrombus formation. In this work, the blood coagulation analysis was carried out in a 96-well irradiated polystyrene culture plate to exclude the effect of the plate surface on coagulation. The clot masses upon contact with microparticles at a concentration of 1 mg/mL and lower (not shown in the graph) were comparable to or inferior to the control sample without the addition of microparticles (Figure 6).

With an increase in the concentration of P(3HB-3HV-3HHх) and chitosan-modified P(3HB-3HV-3HHх) microparticles, the clot masses significantly exceeded the control values. At the same time, no reliable differences were recorded between P(3HB-3HV-3HHх) and chitosan-modified P(3HB-3HV-3HHх) microparticles (Figure 6).

Overall, the results indicate that adding chitosan to the surface of P(3HB-3HV-3HHx) microparticles has a positive effect on their hemolytic activity. At the same time, the results of thrombogenic activity allow us to conclude that P(3HB-3HV-3HHx) and chitosan-modified P(3HB-3HV-3HHx) microparticles are suitable as hemostatic agents for topical use.

### Biocompatibility of microparticles in epithelial cell culture

**Figure 7.**
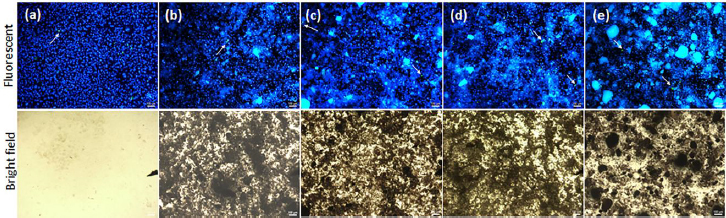
Photo of double fluorescent staining living and dead HaCaT cells (NucBlue^®^ Live staining - blue, live, and NucGreen^®^ Dead staining - green, dead (shown by arrows)) cultured on P(3HB-3HV-3HHх) and chitosan-modified P(3HB-3HV-3HHх) microparticles in comparison with the control (culture plate); the concentration of microparticles was 20 mg/mL: (a) control; (b) P(3HB-3HV-3HHx); (c) P(3HB-3HV-3HHx)-CS_1_; (d) P(3HB-3HV-3HHх)-CS_2_; (e) P(3HB-3HV-3HHx)-CS_3_. 2-day culture. The decoding of analyzed microparticles is presented in Table 2. Bar 100 microns

High biocompatibility of PHAs products is due to the fact that monomers - 3-hydroxybutyric acid are natural metabolites of cells and tissues of organisms and do not cause sharp tissue incorporation and, consequently, a pronounced inflammatory reaction [49,50]. The results of the study of the biological compatibility of P(3HB-3HV-3HHх) and chitosan-modified P(3HB-3HV-3HHх) microparticles in the culture of human skin epithelium – HaCaT keratinocytes are presented in Figure 7.

**Figure 8.**
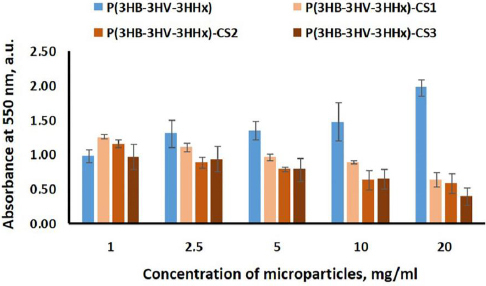
Relative number of HaCaT cells cultured on P(3HB-3HV-3HHх) and chitosan-modified P(3HB-3HV-3HHх) microparticles to the control (culture plate) according to the results of the MTT test. The decoding of analyzed microparticles is presented in Table 2. Data are presented as mean value ± standard deviation, *n* = 6.

The analysis of microphotographs allows us to conclude that the microparticles do not have a negative effect; the cells demonstrate high viability. Single dead cells are noted. The highest viability was noted on the sample from P(3HB-3HV-3HHх), close to 99%. The viability of cells during cultivation in the presence of chitosan-modified microparticles was about 95%; at the same time, the highest number of dead cells was noted on the sample with the maximum chitosan content - P(3HB-3HV-3HHх)-CS3. The results of fluorescent staining are consistent with the measurement of metabolic activity obtained in the MTT test (Figure 8). An increase in the concentration of P(3HB-3HV-3HHх) microparticles led to a 2-fold increase in metabolic activity, most likely due to cell migration and growth on the developed surface of the particles.

The literature describes cell growth on the surface of polymer particles made from PHAs [10,52]. When chitosan was included in the particles, the metabolic activity indices, on the contrary, decreased with increasing particle concentration, which directly correlates with the amount of chitosan. A similar picture was noted by the authors in the paper [41], which showed that the cytotoxicity of composite PLGA microparticles with chitosan depended on the concentration of the introduced particles, with cell survival being about 92 %.

In this study, we found that chitosan-modified P(3HB-3HV-3HHх) microparticles are also biocompatible and observed no toxicity after exposure to the HaCaT cell culture.

### Antibacterial activity of ceftazidime-loaded chitosan-modified P(3HB-3HV-3HHх) microparticles

Polymeric drug delivery systems in the form of micro- and nanoparticles are considered promising agents in the treatment of infectious skin diseases. It has been established that PHAs do not have antimicrobial activity [8]. Therefore, to impart antibacterial properties, it is necessary to introduce various antimicrobial drugs into the composition of polymer products made of PHAs.

It has been shown that the biocidal activity of chitosan is associated with its polycationic structure and the ability to bind to negatively charged surface structures of cells. The first target of chitosan in the case of gram-negative bacteria is lipopolysaccharide, which is negatively charged and is part of the outer membrane. In gram-positive bacteria, the main target for chitosan may be teichoic acids, which are negatively charged by numerous phosphoric acid residues. In both cases, such interaction disrupts the normal functioning of the cell's metabolic processes with the external environment, changing the permeability of the cytoplasmic membrane and resulting in an increased outflow of substances from the cell [53].

In this work, we investigated the antibacterial properties of ceftazidime-loaded chitosan-modified P(3HB-3HV-3HHх) microparticles using model strains of gram-positive and gram-negative microorganisms – *St. aureus* and *E. coli*, respectively. These microorganisms are among the causative agents of nosocomial infections that cause severe complications and also demonstrate high rates of antibiotic resistance in recent decades [54].

**Figure 9.**
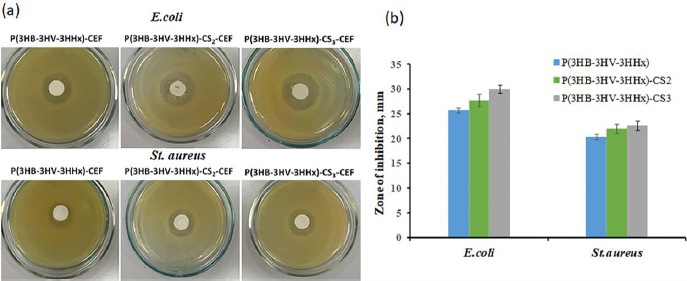
Antimicrobial activity of P(3HB-3HV-3HHх)-CEF and P(3HB-3HV-3HHх)-CS-CEF microparticles with different chitosan content: (a) photo of Petri dishes with inoculations of test cultures of *E. coli* and *St. aureus*; (b) diameter of the inhibition zone. The decoding of analyzed microparticles is presented in Table 2. Data are presented as mean value ± standard deviation, *n* = 3.

The results of the antibacterial activity of ceftazidime loaded in P(3HB-3HV-3HHx) and P(3HB-3HV-3HHx)-CS microparticles are shown in Figure 9.

It was shown that P(3HB-3HV-3HHх)-CS-CEF and P(3HB-3HV-3HHх)-CEF microparticles possess antibacterial activity against both test microorganisms, but to different degrees. The antibacterial effect of ceftazidime was most pronounced in relation to the suppression of gram-negative bacteria *E. coli* when this antibiotic was loaded in chitosan-modified P(3HB-3HV-3HHх) microparticles. Thus, the diameter of the inhibition zone depended on the chitosan content in the microparticles and was 25.7±0.5, 27.7±1.2, 30±0.82 mm, respectively, for P(3HB-3HV-3HHх)-CEF, P(3HB-3HV-3HHх)-CS2-CEF and P(3HB-3HV-3HHх)-CS3-CEF microparticles.

With respect to gram-positive *St.aureus*, the diameter of the bacterial growth inhibition zone was 1.3 times smaller on average. At the same time, microparticles coated with chitosan had a more pronounced antibacterial effect. Thus, the diameter of the inhibition zone for P(3HB-3HV-3HHх)-CS_2_-CEF and P(3HB-3HV-3HHх)-CS_3_-CEF microparticles was comparable and amounted to 22.0±0.9 and 22.6±0.9 mm, respectively. The minimum diameter value of the bacterial growth inhibition zone was noted for P(3HB-3HV-3HHх)-CEF microparticles – 20.3±0.6 mm.

A similar tendency to enhance the antibacterial properties of composite microparticles from P(3HB-3HV) with chitosan containing bacitracin, neomycin or kanamycin was observed in cultures of pathogenic microorganisms *E. coli*, *P. aeruginosa* and *St. aureus* [25].

In general, the obtained results indicate the prospects of using chitosan-modified P(3HB-3HV-3HHх) microparticles containing an antibiotic to suppress the development of pathogenic microorganisms. The results of inhibition of the growth of *E. coli* and *St. aureus* showed that chitosan-modified PHAs microparticles can enhance the effect of the deposited antibiotic and have a combined antibacterial effect compared to PHAs microparticles without modification of the polymer base.

## Conclusions

Microparticles from polymers synthesized on different types of carbon substrates were obtained and studied. The use of oleic acid as a carbon substrate ensured the synthesis of a three-component copolymer P(3HB-3HV-3HHx), which allows obtaining more homogeneous small-sized microparticles. Using a modified emulsification method, chitosan-modified P(3HB-3HV-3HHx) microparticles containing ceftazidime were obtained. The properties of microparticles were studied and the influence of the composition of the polymer base and the content of chitosan on the dimensional characteristics, zeta potential and the efficiency of drug encapsulation into microparticles was shown. The results of the study of the kinetics of ceftazidime release from chitosan-modified microparticles into a model medium showed the suitability of microparticles for the deposition and long-term delivery of drugs, since their release in vitro is gradual and corresponds to the Higuchi and Korsmeyer-Peppas model, has a diffusion nature and obeys Fick's laws. It was shown that chitosan-modified P(3HB-3HV-3HHх) microparticles are also biocompatible and observed no toxicity after exposure to the HaCaT cell culture. Moreover, for chitosan-modified P(3HB-3HV-3HHх) microparticles, hemolysis was not detected over the entire range of experimental concentrations. Chitosan-modified microparticles enhanced the antibacterial activity of ceftazidime, being effective against gram-negative and gram-positive bacteria. Thus, the developed chitosan-modified PHA microparticles can be recommended as promising candidates for alternative antibacterial strategies.
